# Haemodynamic activity characterization of resting state networks by fractal analysis and thalamocortical morphofunctional integrity in chronic migraine

**DOI:** 10.1186/s10194-020-01181-8

**Published:** 2020-09-14

**Authors:** Camillo Porcaro, Antonio Di Renzo, Emanuele Tinelli, Giorgio Di Lorenzo, Vincenzo Parisi, Francesca Caramia, Marco Fiorelli, Vittorio Di Piero, Francesco Pierelli, Gianluca Coppola

**Affiliations:** 1 Institute of Cognitive Sciences and Technologies (ISTC), National Research Council (CNR), Via Palestro 32, I-00185 Rome, Italy; 2 Centre for Human Brain Health and School of Psychology, University of Birmingham, Birmingham, UK; 3 S. Anna Institute and Research in Advanced Neurorehabilitation (RAN), Crotone, Italy; 4 Department of Information Engineering, Università Politecnica delle Marche, Ancona, Italy; 5 Research Center for Motor Control and Neuroplasticity, KU Leuven, Leuven, Belgium; 6 IRCCS Fondazione Bietti, Rome, Italy; 7 Department of Human Neurosciences, Sapienza University of Rome, Rome, Italy; 8 Laboratory of Psychophysiology and Cognitive Neuroscience, Department of Systems Medicine, University of Rome Tor Vergata, Rome, Italy; 9 IRCCS Fondazione Santa Lucia, Rome, Italy; 10 Department of Medico-Surgical Sciences and Biotechnologies, Sapienza University of Rome Polo Pontino, Latina, Italy; 11 IRCCS – Neuromed, Pozzilli, (IS) Italy

**Keywords:** Resting state networks (RSN), Functional magnetic resonance imaging (fMRI), Diffusion tensor imaging (DTI), Fractal analysis (FA), Higuchi’s fractal dimension (FD), Migraine and chronic

## Abstract

**Background:**

Chronic migraine (CM) can be associated with aberrant long-range connectivity of MRI-derived resting-state networks (RSNs). Here, we investigated how the fractal dimension (FD) of blood oxygenation level dependent (BOLD) activity may be used to estimate the complexity of RSNs, reflecting flexibility and/or efficiency in information processing in CM patients respect to healthy controls (HC).

**Methods:**

Resting-state MRI data were collected from 20 untreated CM without history of medication overuse and 20 HC. On both groups, we estimated the Higuchi’s FD. On the same subjects, fractional anisotropy (FA) and mean diffusivity (MD) values of bilateral thalami were retrieved from diffusion tensor imaging and correlated with the FD values.

**Results:**

CM showed higher FD values within dorsal attention system (DAS) and the anterior part of default-mode network (DMN), and lower FD values within the posterior DMN compared to HC. Although FA and MD were within the range of normality, both correlated with the FD values of DAS.

**Conclusions:**

FD of DAS and DMN may reflect disruption of cognitive control of pain in CM. Since the normal microstructure of the thalamus and its positive connectivity with the cortical networking found in our CM patients reminds similar results obtained assessing the same structures but with the methods of neurophysiology, in episodic migraine during an attack, this may be yet another evidence in supporting CM as a never-ending migraine attack.

## Introduction

Up to 3 % of migraines evolve from episodic to chronic annually [1]. It is common agreement that sensitization at the third-order thalamic neurons [2] and at the cortical level [3] drives the functional and clinical changes accompanying migraine chronification. One of the aspects of the migraine brain most explored with functional Magnetic Resonance Imaging (fMRI) is functional activity at rest so called resting-state networks (RSNs). It can capture the macroscopic spatial dynamics of the blood oxygenation level dependent (BOLD) signal of the brain, which is the basis to form networks [4]. Several research groups have detected alterations in the dynamics of different cortical networks in chronic migraine patients [5]. None of them have investigated yet the integrity of the thalamo-cortical network activity in patients with chronic migraine (CM).

Specifically, we aimed to investigate how the dynamics of BOLD activity at rest can be used to differentiate RSNs in CM patients compared to healthy controls (HC).

However, despite the fact that linear methods being predominantly used in characterizing brain oscillations in both healthy and pathological conditions, linear analysis may not be suitable to describe the irregular and non-periodic patterns recorded by electrophysiological and neuroimaging techniques [6, 7].

To this end, we characterized the specific BOLD signature of each RSN, using a complexity measure called fractal dimension (FD) [8] that has advantages over classical linear methods such as the well-known fast Fourier transformation (FFT) that are best suited to conditions where the analysed signals are stationary. We further searched for correlations with microstructure of the thalamus, quantified by acquiring water diffusion metrics. These morpho-functional measures were previously found to be related in episodic migraine [9, 10].

FD is a general measure of complexity derived from chaos theory, based on the fact that a simple process that is repeated endlessly becomes a very complex process, which is the basis for the description of fractals in nature [11]. These complex processes of interactions cause a pattern in brain activity that is self-similar over different spatial and temporal scales. In other words, neural activity shows similar features over and over again on a scale-free basis [12, 13]. Knowing that FD is an accurate numerical measure no matter what the properties (stationary, nonstationary, deterministic or stochastic) of the analysed signal, it is reasonable to accept this advantage over widely used FFT linear method [14]. In addition, recent evidence has demonstrated that in many cases brain signals considered as belonging to a frequency-defined class of brain rhythms do not represent sustained oscillations, but rather brief bouts of activity that are repeated intermittently (i.e., non-rhythmic) [15, 16]. Recognition that physiological time series contain “hidden information” that might be captured by non-linear methods such as fractal analysis, may provide crucial and so far overlooked physiological information in healthy and pathological conditions [17–20]. However, while this type of nonlinear approaches is no longer an issue in the domain of EEG, there are just few applications on the analysis of micro [21] and macro [22, 23] structural neuroimaging data. To the best of our knowledge, there are no MRI studies using FD to determine the spatio-temporal dynamics of the complexity of independent brain networks. Moreover, there are no reports inferring thalamo-cortical network connectivity by correlating the complexity of functional networks with thalamic microstructural metrics obtained by means of diffusion tensor imaging. The thalamic microstructure has been found altered in patients with episodic migraine [9, 24–26] and the thalamus has been considered an important structure in the process of migraine chronification and its related clinical manifestation, i.e. widespread cutaneous allodynia [2]. Despite the uttermost importance thus to gather more data about thalamic microstructure when patients evolve from episodic to CM and to verify its relationship with cortical functional networks at rest, none has done this yet.

Here, we inferred thalamo-cortical networks activity by investigating thalamic microstructure, by means of diffusion tensor imaging (DTI), and independent cortical networking taking the advantages of the innovative non-linear approach of FD analysis in a group of CM patients devoid of medication overuse and without prophylaxis.

## Materials and methods

### Participants

In accordance with the diagnostic criteria of the International Classification of Headache Disorders (ICHD-III), 20 patients with chronic migraine (code 1.3; 14 female and 6 male) were prospectively enrolled (Table 1). All enlisted patients had a clear history of episodic migraine without aura (code 1.1), but not a history of excessive use of symptomatic medications. None of the patients had been on prophylactic therapy in the last 3 months. All but 2 patients who complained of mild headache were recorded during the pain-free period. This study is part of a more comprehensive one in which the same patients underwent multiple neuroimaging tests during the same experimental session. The healthy subjects in this study were published elsewhere [27–29]. The criteria for exclusion were the presence of neurological comorbidities other than migraine, obvious psychiatric disorders, endocrinological disorders, autoimmune or connective tissue disorders, and arterial hypertension. Between one patient and another, 20 healthy controls (HC, 13 women and 7 men) were scanned without any personal or family history of migraine or another primary headache, and any other manifest medical condition. All enrolled women were scanned outside the days of the menstruation (at an average of 17.8 for HC and 18.1 for CM days after the 1st day of the last menstruation). All recordings were conducted in the afternoon, from 4 p.m. to 7 p.m. All participants in the study were informed of its purpose, after which they signed an informed consent. The study was approved by the ethical committee of the Sapienza University of Rome.

**Table 1:** Clinical and demographic data from patients with chronic migraine (CM) and healthy controls (HC)

	Healthy Controls (HC; *n* = 20)	Chronic Migraine (CM; *n* = 20)
**Sex (female/male)**	13 (65%) / 7 (35%)	14 (70%) / 6 (30%)
**Age (years)**	28.75 ± 3.89	31.95 ± 9.88
**Days with headache/month (number)**		23.0 ± 6.8
**Disease duration (year)**		15.0 ± 13.1
**Severity of headache (0–10)**		7.6 ± 1.6
**Duration of the chronic headache phase (months)**		17.1 ± 29.3
**Tablet intake/month (number)**		3.0 ± 3.2

Gender is expressed as frequency (percentage), other data are expressed as mean ± SD

### Data acquisition and preprocessing

MRI data were obtained on a Siemens 3 T Verio scanner using a 12-channel head coil. Structural anatomic scans were performed using a T1-weighted sagittal magnetization-prepared rapid gradient echo (MPRAGE) series (TR: 1900 ms, TE: 2.93 ms, 176 sagittal slices, 0.5 × 0.5 × 1 mm^3^ voxels). To ruling out sub-clinical or other pathologies, we acquired an interleaved double-echo Turbo Spin Echo sequence proton density and T2-weighted images (repetition time: 3320 ms, echo time: 10/103 ms, matrix: 384 × 384, field of view: 220 mm, slice thickness: 4 mm, gap: 1.2 mm, 50 axial slices). Functional MRI data were obtained using T2*-weighted, echo-planar imaging (TR: 3000 ms, TE: 30 ms, 40 axial slices, 3.906 × 3.906 × 3 mm, 150 volumes). For all the sequences but echo-planar bold, to accelerate fMRI acquisitions and minimize distortions, the actual EPI uses multiband sequences with simultaneous echo refocusing and parallel imaging (called Generalized Autocalibrating Partially Parallel Acquisition, GRAPPA, by Siemens). Functional resting scans lasted 7 minutes and 30 s, during which participants were instructed to relax, avoid motion, and keep their eyes closed, but not to fall asleep.

Functional MRI data preprocessing was carried out using SPM12 software (http://www.fil.ion.ucl.ac.uk/spm/) implemented in MATLAB (version R2016b, MathWorks, Inc., Natick, MA, USA). Data were realigned to the first volume to correct for head motion using a 6-parameter rigid body process and resliced by a cubic spline interpolation. The structural (T1–MPRAGE) and functional data were coregistered for each participant dataset. Normalization procedure transformed structural and realigned EPI images into a common stereotactic space based on Talairach and Tournoux, resampled by 3 mm on each direction. Finally, the spatially normalized functional images were smoothed isotropically at 8x8x8 mm., segmented into grey matter, white matter and CSF using the Tissue Probability Map template, normalized into standard Montreal Neurological Institute space using nonlinear transformations and smoothed with a Gaussian smoothing kernel of 8 mm full-width at half-maximum.

Diffusion tensor imaging (DTI) was acquired by using single shot echo-planar imaging, with an 12–channel head coil (TR 12200 ms, TE 94 ms, 72 axial slices, 2 mm thickness, isotropic voxels), using SPAIR (Spectral Attenuated Inversion Recovery) as fat suppression technique. Images from the same participants and during the same session were obtained with diffusion gradients applied along 30 non-collinear directions, effective b values of 0 and 1000 s/mm^2^ were used.

We used FSL 6.0 software package (FMRIB Image Analysis Group, Oxford, England; https://fsl.fmrib.ox.ac.uk/fsl/fslwiki) to process DTI data. The FSL Diffusion Toolbox (FDT) was used to correct susceptibility induced distortions [30], eddy currents [31] and motion artifacts [32], while the brain extraction tool (BET) was used to create brain masks from the b0 image of each participant [33]. An automated quality control framework was used to assess diffusion MRI data [34].

### fMRI data analysis

After data preprocessing, resting state data of all participants as a concatenated groups (healthy controls, HC, and chronic migraine, CM) were analysed using spatial independent component analysis (ICA) as implemented in the Group ICA of fMRI Toolbox (GIFT; http://trendscenter.org/software/gift/) to decompose the data into functional networks that exhibited a unique time course profile. Two data reduction steps were carried out using principal component analysis, subject-specific and group-level steps. Firstly, subject-specific data were reduced to 30 components and subject-reduced data were concatenated across time. Secondly, at group level, data were reduced into 20 group independent components (ICs) with the expectation-maximization algorithm, included in GIFT [35]. We propose using standard information theoretic methods for estimating the number of components from the aggregate data set. These methods make a decision based upon the complexity or information content of the data. The number of ICs was estimated using the minimum description length (MDL) criterion [36, 37]. In our specific case 20 independent components (ICs) were indicated to be estimated. Subject-specific spatial maps and time courses were obtained using the back-reconstruction approach (GICA) [36].

**Fig. 1:**
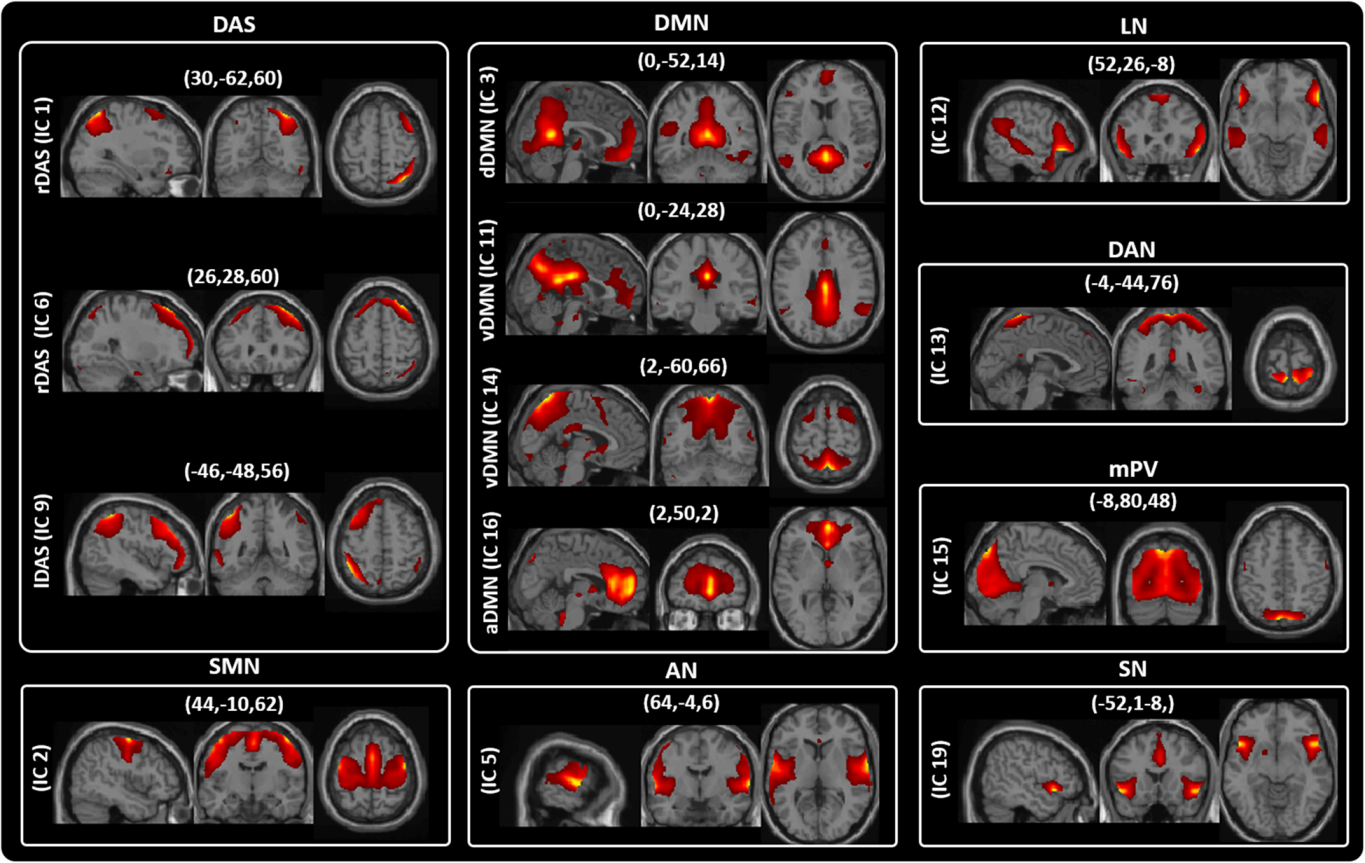
Resting State Networks (RSNs) identified by GIFT. Thirteen spatial maps divided into eight functional networks: 1) dorsal attention system (DAS: IC1 - rDAS, IC6 - rDAS and IC9 - lDAS); 2) sensorimotor (SMN: IC2); 3) default mode (DMN: IC3 - dDMN, IC11 and IC14 - vDMN, IC16 - aDMN), 4) Auditory (AN: IC5), 5) Language (LN: IC12), 6) Dorsal Attention (DAN: IC13), 7) medial Primary Visual (mPV: IC15), 8) salience (SN: IC19) networks based on their anatomical view

From the 20 ICs, we identified the relevant RSNs by applying a previously described procedure [35]. We first manually confirmed if the peak activation coordinates were located primarily in grey matter, showing low spatial overlap with vascular, ventricular, edge regions corresponding to artefacts [35]. This process resulted in thirteen meaningful ICs that we sorted into eight functional networks, based on the spatial correlation between independent components and the template provided by GIFT Toolbox [35] as follow (Fig. 1 and Table S1): dorsal attention system (DAS – IC1, IC6 and IC9); sensorimotor network (SMN – IC2); default mode network (DMN – IC3, IC11, IC14, IC16); Auditory Network (AN – IC5); Language Network (LN – IC12); Dorsal Attention Network (DAN – IC13); medial Primary Visual (mPV – IC15) and salience network (SN – IC19).

### Characterization of the BOLD RSNs by Higuchi’s fractal dimension

Higuchi’s FD [8] is a nonlinear measure of waveform complexity in the time domain. Discretized functions or signals can be analyzed as segment of data X (1), X (2), …, X(N), where N is the total number of samples. From the starting time sequence, a new self-similar time series ${X}_m^k$ can be calculated as:

$$ {X}_m^k:x(m),x\left(m+k\right),x\left(m+2k\right),\dots, x\left(m+\mathit{\operatorname{int}}\left(\frac{N-k}{k}\right)k\right) $$

for *m = 1, 2, …, k* where *m* is the initial time; *k* is the time interval, *k = 1, 2, …, k*_*max*_; *k*_*max*_ is a free parameter, and *int(r)* is the integer part of the number *r*.

The length, L_m_(k), of each curve X^k^_m_ is calculated as:

$$ {L}_m(k)=\frac{1}{k}\left[\sum \limits_{i=1,\operatorname{int}\left(\frac{N-m}{k}\right)}\left|X\left(m+ ik\right)-X\left(m+\left(i-1\right)k\right)\right|\cdot \frac{N-1}{\operatorname{int}\left(\frac{N-m}{k}\right)}\right] $$

where N is the length of the original time series X and (N − 1)/{int[(N − m)/k]k} is a normalization factor. L_m_(k) was averaged for all *m* forming the mean value of the curve length *L(k)* for each *k = 1, …, k*_*max*_ as:

$$ L(k)=\frac{\sum \limits_{m=1}^k{L}_m(k)}{k} $$

An array of mean values *L(k)* was obtained and the FD was estimated as follow:

*FD = ln(L(k))/ln (1/k)* for *k = 1, 2, …, k*_*max*_.

In practice, the original curve or signal can be divided into smaller parts with or without overlap, called “windows”. Then, the method for computing FD should be applied to each window where N should be seen as the length of the window. In that case, FD values are calculated for each window, with or without overlap. Individual FD values can be averaged across all windows for the entire curve, and the mean FD value can be used as a measure of curve complexity.

Here, using the single-subject IC time courses for each RSN, we calculated FD in non-overlapped time windows of 150 s (corresponding to 50 of our fMRI volumes). The choice of the free parameter *k* has a crucial role in FD estimation. For each window we estimated twenty-four values of FD for *k* = 2, …, 25. The value 25 was equal to half of the samples within our 50 volumes window (i.e. 150 s). *k*_*max*_ is equal to half of the window length the maximum length that can be chosen. There were three windows within our 150 volume scans, therefore we estimated three measures of FD at each value of *k* (e.g. FD_2_, FD_3_, FD_4,_ …., FD_24_). These three measures were averaged to give one mean value of FD for each *k*, for each subject [14, 20, 38]. The process was then repeated for every subject and every RSN (in Porcaro and colleagues [39] it is shown in detail the procedure and additional analyses demonstrating that the FD measurements were not dependent on the choice of window length or overlapping windows).

### Diffusion tensor imaging (DTI) analysis

The FSL toolbox DTIFIT fits the pre-processed image based on a diffusion tensor model to yield FA and MD. For each subject, two regions of interest (ROI) were created, which cover the total left and right thalamus on each slice. The medial boundaries were identified on each slice using the Cerebral Spinal Fluid (CSF) as limits, lateral limits were also verified using FA maps to exclude the internal capsule. Mean FA and MD values in each region for every subject were determined by averaging those voxels in the ROI.

### Statistical analysis

Kolmogorov-Smirnov test for normality indicated that FD values of all the RSNs and the FA and MD values for left and right thalamus did not differ from a Gaussian distribution (consistently, *p* > 0.200).

Repeated-measures analysis of variance (rm-ANOVA) was performed on the FD values to investigate the interaction effect *GROUPs × ICs* (the two *GROUPs* as between-subject factor: *CM* vs. *HC*; the thirteen *ICs* as within-subjects factor: *IC1* vs. *IC2* vs. *IC3* vs. *IC5* vs. *IC6* vs. *IC9* vs. *IC11* vs. *IC12* vs. *IC13* vs. *IC14* vs. *IC15* vs. *IC16* vs. *IC19*). Repeated-measures multivariate analysis of variance (rm-MANOVA) was carried out on FA and MD values to explore the interaction effect *GROUPs × SIDEs* (as before, the two *GROUPs* as between-subject factor: *CM* vs. *HC*; the two *SIDEs* as within-subjects factor: *left thalamus* vs. *right thalamus*). In both analyses, univariate ANOVA results were analyzed only if the Wilks’ Lambda multivariate significance criterion was achieved. The sphericity of the covariance matrix was verified with the Mauchly sphericity test. In the case of violation of the sphericity assumption, the Greenhouse–Geisser epsilon adjustment was used. Cohen’s *d* (and its 95% confidence intervals, CI_95_) was used as measure of effect size.

Pearson’s correlation test was performed between the FD values for each IC, the FA and MD values for left and right thalamus and clinical variables (including: severity of headache attacks, ranging 0 to 10; duration of migraine history, in years; number of monthly days with headache; duration of the chronic phase, expressed in months; monthly number of acute medications).

Significance threshold was set at *p*-value < 0.05.

## Results

Demographic characteristics of CM and HC and clinical features of CM are summarized in Table 1. No significant difference emerged between CM and HC in gender (${\chi}_1^2$ = 0.114, *p* = 0.736) and age (*t*_38_ = 1.348, *p* = 0.186).

In CM, there were not white matter lesions. Mean and standard deviation of FD values as well as thalami FA and MD values for CM and HC are reported in Table 2.

**Table 2:** Mean and standard deviation (in brackets) for the bilateral thalami fractional anisotropy (FA) and mean diffusivity (MD) together with fractal dimension (FD) values for IC1, IC14 and IC16

HC						
Left		Right		FD		
**FA**	**MD**	**FA**	**MD**	**IC1**	**IC14**	**IC16**
0.34	119	0.33	122	1.840	1.901	1.855
(0.03)	(12.7)	(0.02)	(10.1)	(0.06)	(0.05)	(0.06)
**CM**						
**Left**		**Right**		**FD**		
**FA**	**MD**	**FA**	**MD**	**IC1**	**IC14**	**IC16**
0.34	119	0.33	121	1.883	1.853	1.899
(0.03)	(11.1)	(0.03)	(12.8)	(0.05)	(0.08)	(0.05)

### Characterization of the BOLD RSNs by Higuchi’s fractal dimension

**Fig. 2:**
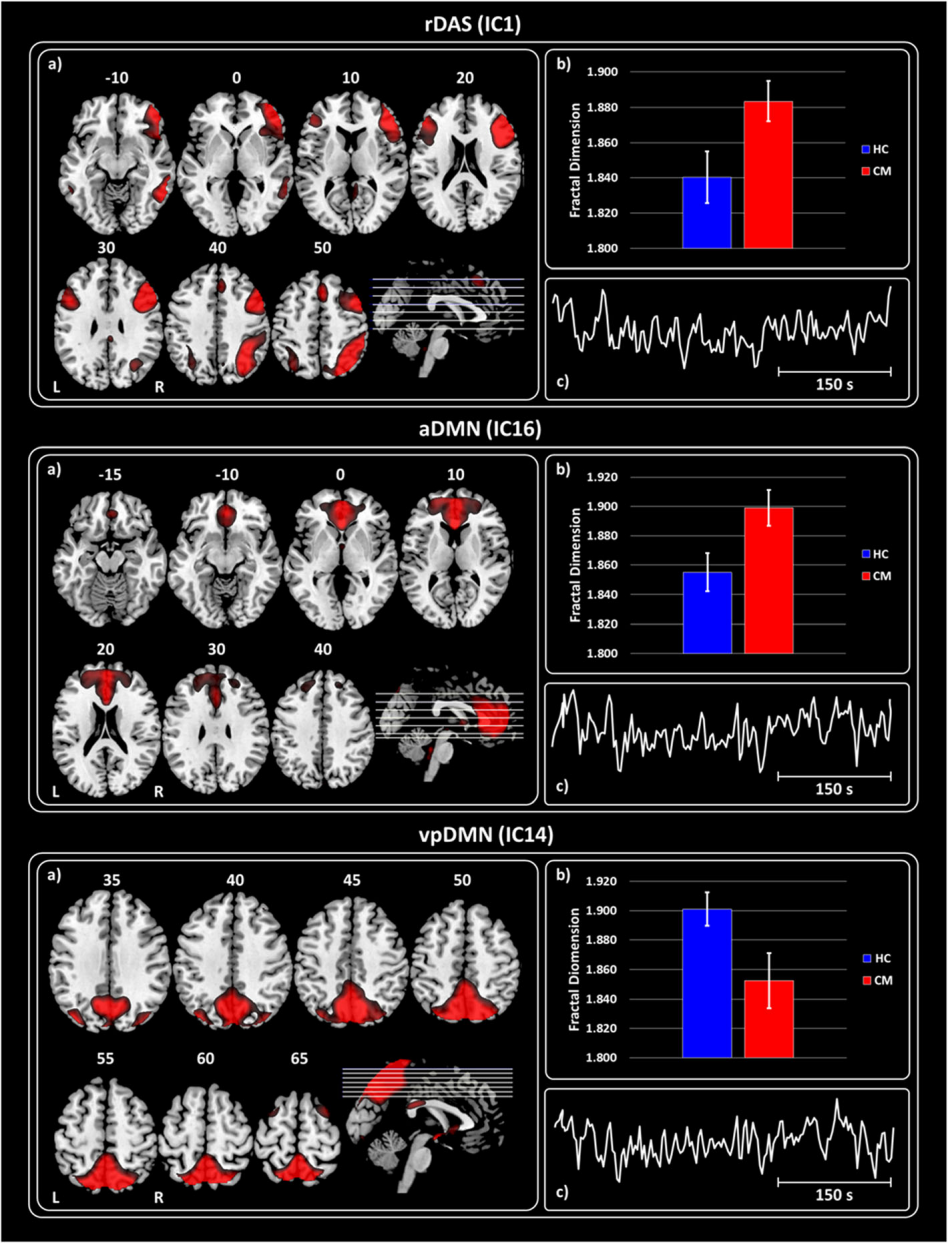
Haemodynamic activity characterization at rest by Fractal Dimension. **For each panel (Upper, Middle and Bottom) – a**) Spatial maps of the IC obtained by GIFT toolbox representing the rDAS, aDMN and vpDMN. **b**) Grand average and standard error for the FD values (k = 12) are shown for both groups HC (blue) and CM (red). **c**) Haemodynamic activity of the IC is shown. **Upper panel** – Shows the results obtained for IC1 representing rDAS. **Middle panel –** Shows the results for IC 16 representing aDMN. **Bottom panel –** Shows the results for IC 14 representing vpDMN. All images have been coregistered into the Montreal Neurological Institute (MNI) space. The numbers above each image refers to the Z coordinate in MNI space

The rm-ANOVA model for FD values revealed that the interaction effect *GROUPs × ICs* was significant (Wilks’ *λ* = 0.434, F_12,27_ = 2.655, *p* = 0.010). Because the sphericity assumption was violated (Mauchly’s W = 0.012, ${\chi}_{77}^2$ = 150.861, *p* < 0.0001), the *ε* adjustment was adopted in the univariate test for repeated measure, which resulted significant (F_12,456_ = 2.700, *ε* = 0.598, p = 0.010). At univariate level, CM differed from HC in FD values of IC1 (F_1,38_ = 6.018, *p* = 0.019), IC14 (F_1,38_ = 5.472, *p* = 0.025) and IC16 (F_1,38_ = 6.751, *p* = 0.013). Mean and standard error (SE) for the significant ICs (CM in red and HC in blue) are shown in Fig. 2. Respect to the HC, higher FD values were observed in CM for IC1 (rDAS) [*d* = 0.80 (0.77, 0.82); Fig. 2 – upper panel] and IC16 (aDMN) [*d* = 0.84 (0.82, 0.87); Fig. 2 – middle panel]. The opposite pattern was observed for IC14 (pDMN) [*d* = − 0.76 (− 0.74, − 0.79); Fig. 2 – bottom panel] with lower FD for the CM respect to the HC.

### Characterization of thalami DTI

The rm-ANOVA model revealed that the interaction effect *GROUPs × SIDEs* was not significant for FA values and for MD values (Wilks’ *λ* = 0.993, F_2,37_ = 0.137, *p* = 0.872). These results indicated that no difference emerged in FA and MD values of left and right thalamus between CM and HC.

### Correlation analysis between FD and thalami DTI

**Fig. 3:**
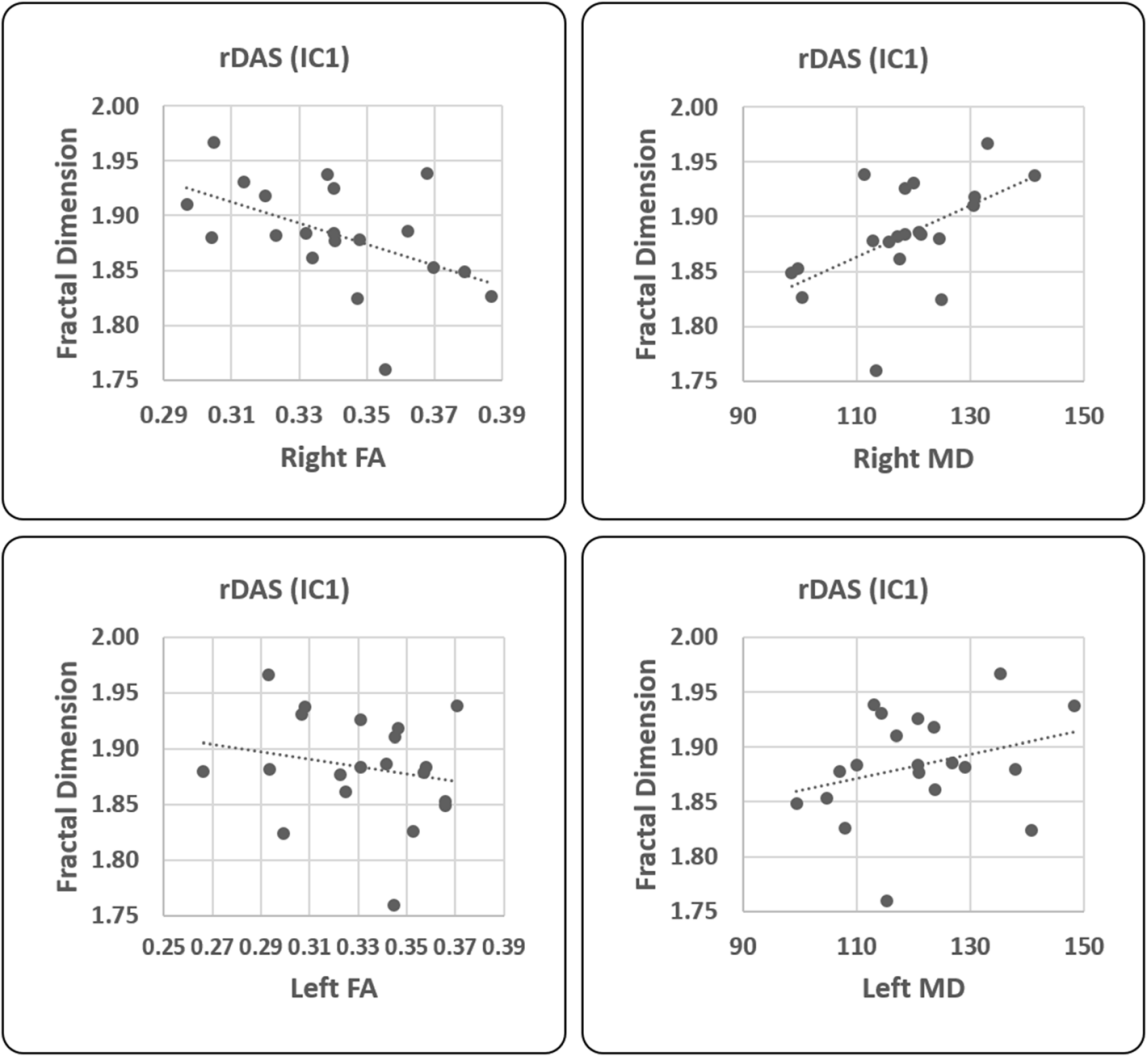
Correlation analysis between FD and thalami values. Pearson’s correlation analysis between FD, right (up row, significant correlation) and left (bottom row, no correlation) for fractional anisotropy (FA, left column) and right mean diffusivity (MD, right column) values

In HC, the FD of IC1 (rDAS) correlated positively with that of IC16 (pDMN, F = 6.29, *p* = 0.023, R2 = 27.01%, R2 adj = 22.72%). There was no significant linear relation between the FD values and FA and MD values. In CM, the FD of IC1 (rDAS) correlated positively with that of both IC14 (pDMN, F = 4.95, *p* = 0.04, R2 = 22.55%, R2 adj = 17.99%) and IC16 (aDMN, F = 6.45, *p* = 0.021, R2 = 27.51%, R2 adj = 23.24%). In CM, the FD values of IC1 (rDAS) correlated negatively with the FA values of right thalamus (r-Thalamus, F = 22.94, *p* < 0.001, R2 = 57.43%, R2 adj = 54.93%); Fig. 3, left panel) and positively with the MD values of right thalamus (r-Thalamus, F = 5.77, *p* = 0.028, R2 = 25.35%, R2 adj = 20.96%); Fig. 3, right panel). No other significant relation emerged between the FD values of other ICs and FA and MD values of thalami. Clinical variables did not correlate with any of the FD values of ICs and FA and MD values of thalami.

## Discussion

In this study, we have investigated how Higuchi’s FD as a measure of multi-scale signal complexity can be used to characterize and differentiate the resting BOLD fMRI signal in HC and CM groups. Linear methods commonly used for signal analysis, such as the well-known Fast Fourier Transformation (FFT) and wavelet transformation (WT), are good choices if the analyzed signals are stationary. However, neurophysiological processes are generally nonstationary and nonlinear by nature. Knowing that FD is an accurate numerical measure no matter what the properties (stationary, nonstationary, deterministic or stochastic) of the analyzed signal, it is reasonable to accept this advantage over widely used linear methods [14]. Among all the proposed FD algorithms, Higuchi’s methods [8] is considered to be the most accurate to estimate FD [40].

The key novel results of this fMRI study can be summarized as follows: a) FD is able to characterize haemodynamic activity also from fMRI at rest, b) compared to HC, CM patients showed a peculiar within networks complexity estimated by FD, and c) although basic DTI metrics did not differ between groups, in patients with CM, right FA and MD were correlated with the FD of the homolateral DAS.

Recently, the application of fractal analysis was extended to the understanding of the underlying dynamics of the micro [21] and macro [22, 23] structure of the brain. Some researchers calculated FD from integrated BOLD signals obtained from resting state functional MRI, but using a voxel-based approach [22, 23]. Here we showed, for the first time, that Higuchi’s fractal analysis can be estimated also from resting state data of different networks identified using group independent component analysis.

Despite the appropriate interpretation of such FD metric remains for the most part incomplete, loss or gain in complexity in brain activity means that the neural system of the brain is less or more flexible and/or efficient in information processing [41]. The complexity of the brain is the result of the interaction between a huge number of brain cells or neuronal structures correlating at long-range and operating at multiple dimensional levels such as space and time. Nonetheless, the complexity of the brain activity further depends on feedback/feedforward information’s flow from the periphery. This level of complexity is extremely variable, since it might increase or decrease depending on the change required of the intrinsic dynamic supporting the measured process, and, furthermore, according to age and pathology [41]. In our CM patients, we observed that two cortical networks, DAS and DMN, showed significant FD differences compared to HC.

The default mode brain network (DMN), most active during internally focused tasks, involves several regions including the medial prefrontal cortex (MPFC), posterior cingulate cortex, precuneus (PCu), and inferior parietal lobules (IPLs) [42]. It was previously shown that DMN can be decomposed in different interacting subnetworks in which anterior or posterior areas can be predominantly included [43]. Coherently, here we identified two subnetworks of the DMN with a significant different FD: the anterior which prominently includes MPFC, and a posterior DMN which includes PCu and IPLs.

Several studies in episodic migraine concordantly found that DMN is less connected between attacks of migraine without aura [44, 45]. Whereas, the intrinsic functional connectivity of the DAS was reduced [46] in episodic migraine interictally and less connected with the DMN [9]. Key regions of the DAS are bilateral frontal eye fields and the bilateral superior parietal gyrus/intraparietal sulcus. They are responsible for top-down cognitive selection of relevant sensory information, multimodal stimulus processing – with a preference for the visual ones – and preparation of responses or action selection [47].

Here, we observed higher FD in DAS and aDMN – covering the anterior subsystem of the DMN – intrinsic functional connectivity, and lower complexity in pDMN – covering the posterior subsystem of the DMN – in CM patients compared to HC. Moreover, we found positive correlations between FD values of DAS and DMN in both groups of participants, but more consistently in CM than in HC, since the former was evident both for aDMN and pDMN, not just for the pDMN as for the latter.

It is well-known that DMN and DAS, devoted to internally and externally processing of goal-directed cognition respectively, are separate and functionally competitive networks [47]. Resting-state data systematically showed that spontaneous DMN activity is anticorrelated to that of DAS, the former being deactivated by attention-demanding tasks [48]. Nonetheless, several studies showed that when the context enhances cognitive loads such as during pain demanding attention – viewed as the focusing of cognitive resources on a specific stimulus [49] –, the DMN is more intensely activated, rather than deactivated [50], and that acute and chronic pain conditions lead to the appearance of a strong correlation between DMN and DAS [51]. These findings are supported by our previous observation in the same group of subjects of a stronger correlation between DMN and DAS in CM patients [28]. However, in contrast with the higher complexity of functional connectivity we found within DAS and the aDMN, we discovered a lower fractal dimensionality within the pDMN of our CM patients. We know from analyses employing a graph theoretical approach that PCu, a hub of the pDMN [52], plays a role as connector area between the DAS and DMN [53], and that its dysfunction could contribute to the emergence of functional brain disorders [54]. Therefore, we cannot exclude that a low dimensionality, i.e. less efficiency in information processing, within the pDMN could explain the lack of anticorrelation between DAS and DMN in response to chronic headache.

Finally, we postulate that the pattern of connectivity and fractal dimensionality presently observed can be the result of the inefficiency of the brain of chronic migraineurs to reserve cognitive resource to pain, i.e. to cope with the chronic recurrence of headache, favouring internal mentation process.

The analysis of microstructural data does not reveal a significant difference between the metrics of diffusivity of patients and those of HC. This is the first study exploring the microstructure of the thalami in CM treatment-free and without a history of medication overuse. Previous neurophysiological studies showed sensitization of visual and somatosensory cortical responses with normal thalamo-cortical loop activation and lateral inhibition in CM. Since this electrophysiological pattern is quite similar to that derived from recordings of ictal episodic migraineurs [3, 55], it was hypothesized that CM is a condition of “never-ending migraine attack”. Coherently, here we found diffusive metrics within the normal ranges in CM, quite similar to those obtained during episodic migraine attacks [10]. These results are in contrast with the abnormal microstructural results obtained in episodic migraine between attacks [9]. Nonetheless, despite these normal results, we found that the more the FD of rDAS the lowed the FA and the higher the MD of right thalamus. Again, similar correlation was previously detected by the help of clinical neurophysiology. In a group of patients with de-novo CM, the higher the amplitude of the somatosensory thalamocortical loop activity, the higher the primary cortical activation in CM and in episodic migraine during an attack, but the same correlation was absent in HC [3]. Whether the correlation between the thalamic microstructure and the complexity of the cortical network is driven primarily by the increase efficiency in cortical information processing, or is secondary to sensitization of the third-order thalamic neurons receiving convergent input from the peripheral ophthalmic division of the trigeminal nerve remains to be determined. Our study has important limitations. Firstly, our sample is relatively low, although statistical analysis gives clearly robust significant results. In the future, it will be important to confirm these results in a larger population of subjects. Secondly, we did not verify cognitive performances and we did not quantitatively score mood and anxiety in our subjects. It has previously shown in fact that bilateral PCu is deactivated during anticipatory anxiety or pain expectation [51].

## Conclusion

Here, we probe that Higuchi’s FD can be used to estimate complexity within networks activity extracted from the MRI at rest using an independent component analysis. The application of this analysis on CM patients demonstrated that an aberrant increase in complexity within the DAS and aDMN and a lower complexity within the pDMN compared to HC. This abnormal pattern of FD within DAS and DMN may reflect disruption of cognitive control of head pain. Normal microstructure of the thalamus and its positive connectivity with the cortical networking may be yet another evidence in support of CM as a never-ending migraine attack.

Further studies are needed to verify whether pharmacological or non-pharmacological migraine prevention treatments are able to modulate the cortical networking fractal dimensionality, in parallel with the reduction in the frequency of attacks.

## Supplementary information

**Additional file 1 Table S1.** Spatial correlation with respect to (w.r.t.) template

## Data Availability

Clinical, neurophysiological and statistical data will be available upon request from any qualified investigator.

## Abbreviations

MRI: Magnetic Resonance Imaging
fMRI: Functional Magnetic Resonance Imaging
DTI: Diffusion tensor imaging
CR: Chronic migraine
RSNs: Resting-state networks
FD: Fractal dimension
BOLD: Blood oxygenation level dependent
HC: Healthy controls
FA: Fractional anisotropy
MD: Mean diffusivity
FFT: Fast Fourier transformation
WT: Wavelet transformation
ICHD: International Classification of Headache Disorders
ICA: Independent component analysis
GIFT: Group ICA of fMRI Toolbox
AIC: Akaike’s information criterion
MDL: The minimum description length
ROI: Regions of interest
CSF: Cerebral Spinal Fluid
DAS: Dorsal attention system
SMN: Sensorimotor network
DMN: Default mode network
AN: Auditory Network
LN: Language Network
DAN: Dorsal Attention Network
mPV: Medial Primary Visual
SN: Salience network

## References

[1] Scher AI, Stewart WF, Ricci JA, Lipton RB (2003). Factors associated with the onset and remission of chronic daily headache in a population-based study. Pain.

[2] Burstein R, Jakubowski M, Garcia-Nicas E et al (2010) Thalamic sensitization transforms localized pain into widespread allodynia. Ann Neurol. 10.1002/ana.21994 10.1002/ana.21994 PMC2930514 20582997

[3] Coppola G, Iacovelli E, Bracaglia M (2013). Electrophysiological correlates of episodic migraine chronification: evidence for thalamic involvement. J Headache Pain.

[4] Fox M, Raichle ME (2007). Spontaneous fluctuations in brain activity observed with functional magnetic resonance imaging. Nat Rev.

[5] Filippi M, Messina R (2020). The chronic migraine brain: what have we learned from neuroimaging?. Front Neurol.

[6] Cole SR, Voytek B (2017). Brain oscillations and the importance of waveform shape. Trends Cogn Sci.

[7] Ignaccolo M, Latka M, Jernajczyk W et al (2010) The dynamics of EEG entropy. J Biol Phys. 10.1007/s10867-009-9171-y 10.1007/s10867-009-9171-y PMC2825306 19669909

[8] Higuchi T (1988) Approach to an irregular time series on the basis of the fractal theory. Phys D Nonlinear Phenom. 10.1016/0167-2789(88)90081-4

[9] Coppola G, Di Renzo A, Tinelli E et al (2016) Thalamo-cortical network activity between migraine attacks: insights from MRI-based microstructural and functional resting-state network correlation analysis. J Headache Pain. 10.1186/s10194-016-0693-y 10.1186/s10194-016-0693-y PMC5078119 27778244

[10] Coppola G, Di Renzo A, Tinelli E et al (2016) Thalamo-cortical network activity during spontaneous migraine attacks. Neurology. 10.1212/WNL.0000000000003327 10.1212/WNL.0000000000003327 27742813

[11] Di Ieva A, Esteban FJ, Grizzi F et al (2015) Fractals in the neurosciences, part II: clinical applications and future perspectives. Neuroscientist 21(1):30–43. 10.1177/1073858413513928 10.1177/1073858413513928 24362814

[12] Stam CJ (2005). Nonlinear dynamical analysis of EEG and MEG: review of an emerging field. Clin Neurophysiol.

[13] Buzsáki G, Mizuseki K (2014) The log-dynamic brain: how skewed distributions affect network operations. Nat Rev Neurosci 15(4):264–278. 10.1038/nrn3687 10.1038/nrn3687 PMC4051294 24569488

[14] Kesić S, Spasić SZ (2016). Application of Higuchi’s fractal dimension from basic to clinical neurophysiology: a review. Comput Methods Prog Biomed.

[15] Feingold J, Gibson DJ, Depasquale B, Graybiel AM (2015) Bursts of beta oscillation differentiate postperformance activity in the striatum and motor cortex of monkeys performing movement tasks. Proc Natl Acad Sci U S A. 10.1073/pnas.1517629112 10.1073/pnas.1517629112 PMC4640760 26460033

[16] Lundqvist M, Rose J, Herman P et al (2016) Gamma and Beta bursts underlie working memory. Neuron. 10.1016/j.neuron.2016.02.028 10.1016/j.neuron.2016.02.028 PMC5220584 26996084

[17] Ahmadlou M, Adeli H (2012) Visibility graph similarity: a new measure of generalized synchronization in coupled dynamic systems. Phys D Nonlinear Phenom. 10.1016/j.physd.2011.09.008

[18] Goldberger AL, Amaral LAN, Hausdorff JM (2002). Fractal dynamics in physiology: alterations with disease and aging. Proc Natl Acad Sci U S A.

[19] Porcaro C, Cottone C, Cancelli A (2019). Cortical neurodynamics changes mediate the efficacy of a personalized neuromodulation against multiple sclerosis fatigue. Sci Rep.

[20] Smits FM, Porcaro C, Cottone C (2016). Electroencephalographic fractal dimension in healthy ageing and Alzheimer’s disease. PLoS One.

[21] Krohn S, Froeling M, Leemans A (2019). Evaluation of the 3D fractal dimension as a marker of structural brain complexity in multiple-acquisition MRI. Hum Brain Mapp.

[22] Kiviniemi V, Remes J, Starck T (2009). Mapping transient hyperventilation induced alterations with estimates of the multi-scale dynamics of BOLD signal. Front Neuroinform.

[23] Weber AM, Soreni N, Noseworthy MD (2014). A preliminary study on the effects of acute ethanol ingestion on default mode network and temporal fractal properties of the brain. Magn Reson Mater Physics, Biol Med.

[24] Granziera C, DaSilva AFM, Snyder J (2006). Anatomical alterations of the visual motion processing network in migraine with and without aura. PLoS Med.

[25] Granziera C, Daducci A, Romascano D (2014). Structural abnormalities in the thalamus of migraineurs with aura: a multiparametric study at 3 T. Hum Brain Mapp.

[26] Coppola G, Tinelli E, Lepre C (2014). Dynamic changes in thalamic microstructure of migraine without aura patients: a diffusion tensor magnetic resonance imaging study. Eur J Neurol.

[27] Coppola G, Petolicchio B, Di Renzo A (2017). Cerebral gray matter volume in patients with chronic migraine: correlations with clinical features. J Headache Pain.

[28] Coppola G, Di Renzo A, Petolicchio B (2019). Aberrant interactions of cortical networks in chronic migraine. Neurology.

[29] Coppola G, Di Renzo A, Petolicchio B (2020). Increased neural connectivity between the hypothalamus and cortical resting-state functional networks in chronic migraine. J Neurol.

[30] Andersson JLR, Skare S, Ashburner J (2003). How to correct susceptibility distortions in spin-echo echo-planar images: application to diffusion tensor imaging. Neuroimage.

[31] Andersson JLR, Sotiropoulos SN (2016). An integrated approach to correction for off-resonance effects and subject movement in diffusion MR imaging. Neuroimage.

[32] Jenkinson M, Bannister P, Brady M, Smith S (2002). Improved optimization for the robust and accurate linear registration and motion correction of brain images. Neuroimage.

[33] Smith SM (2002). Fast robust automated brain extraction. Hum Brain Mapp.

[34] Bastiani M, Cottaar M, Fitzgibbon SP (2019). Automated quality control for within and between studies diffusion MRI data using a non-parametric framework for movement and distortion correction. Neuroimage.

[35] Allen EA, Damaraju E, Plis SM (2014). Tracking whole-brain connectivity dynamics in the resting state. Cereb Cortex.

[36] Calhoun VD, Adali T, Pearlson GD, Pekar JJ (2001) Spatial and temporal independent component analysis of functional MRI data containing a pair of task-related waveforms. Hum Brain Mapp. 10.1002/hbm.1024 10.1002/hbm.1024 PMC6871956 11284046

[37] Porcaro C, Zappasodi F, Rossini PM, Tecchio F (2009). Choice of multivariate autoregressive model order affecting real network functional connectivity estimate. Clin Neurophysiol.

[38] Marino M, Liu Q, Samogin J (2019). Neuronal dynamics enable the functional differentiation of resting state networks in the human brain. Hum Brain Mapp.

[39] Porcaro C, Mayhew SD, Marino M et al (2020) Characterisation of Haemodynamic activity in resting state networks by fractal analysis. Int J Neural Syst S0129065720500616. 10.1142/S0129065720500616 10.1142/S0129065720500616 33034533

[40] Esteller R, Vachtsevanos G, Echauz J, Litt B (2001) A comparison of waveform fractal dimension algorithms. IEEE Trans Circuits Syst I Fundam Theory Appl. 10.1109/81.904882

[41] Goldberger AL, Peng CK, Lipsitz LA (2002). What is physiologic complexity and how does it change with aging and disease? Neurobiol. Aging.

[42] Raichle M, MacLeod AM, Snyder AZ (2001). A default mode of brain function. Proc Natl Acad Sci U S A.

[43] Conwell K, von Reutern B, Richter N (2018). Test-retest variability of resting-state networks in healthy aging and prodromal Alzheimer’s disease. NeuroImage Clin.

[44] Tessitore A, Russo A, Giordano A (2013). Disrupted default mode network connectivity in migraine without aura. J Headache Pain.

[45] Yu D, Yuan K, Luo L (2017). Abnormal functional integration across core brain networks in migraine without aura. Mol Pain.

[46] Yang F-C, Chou K-H, Hsu A-L (2018). Altered brain functional Connectome in migraine with and without restless legs syndrome: a resting-state functional MRI study. Front Neurol.

[47] Corbetta M, Shulman GL (2002). Control of goal-directed and stimulus-driven attention in the brain. Nat Rev.

[48] Fox MD, Snyder AZ, Vincent JL (2005). From the cover: the human brain is intrinsically organized into dynamic, anticorrelated functional networks. Proc Natl Acad Sci.

[49] Seminowicz DA, Davis KD (2007). Pain enhances functional connectivity of a brain network evoked by performance of a cognitive task. J Neurophysiol.

[50] Berna C, Leknes S, Holmes EA (2010). Induction of depressed mood disrupts emotion regulation Neurocircuitry and enhances pain unpleasantness. Biol Psychiatry.

[51] Ter Minassian A, Ricalens E, Humbert S (2013). Dissociating anticipation from perception: acute pain activates default mode network. Hum Brain Mapp.

[52] Buckner RL, Andrews-Hanna JR, Schacter DL (2008). The brain’s default network: anatomy, function, and relevance to disease. Ann N Y Acad Sci.

[53] Spreng RN, Sepulcre J, Turner GR (2013). Intrinsic architecture underlying the relations among the default, dorsal attention, and frontoparietal control networks of the human brain. J Cogn Neurosci.

[54] Schmidt SA, Carpenter-Thompson J, Husain FT (2017). Connectivity of precuneus to the default mode and dorsal attention networks: a possible invariant marker of long-term tinnitus. NeuroImage Clin.

[55] Coppola G, Cortese F, Bracaglia M et al (2020) The function of the lateral inhibitory mechanisms in the somatosensory cortex is normal in patients with chronic migraine. Clin Neurophysiol. 10.1016/j.clinph.2020.01.009 10.1016/j.clinph.2020.01.009 32070811

