# Supplementary material for: Haemodynamic activity characterization of resting state networks by fractal analysis and thalamocortical morphofunctional integrity in chronic migraine
Source: J Headache Pain. 2020 Sep 14;21(1):112. doi: 10.1186/s10194-020-01181-8 (PMC7490862; doi:10.1186/s10194-020-01181-8)
Supplement: Supplementary file 1 — Additional file 1 Table S1. Spatial correlation with respect to (w.r.t.) template [file 10194_2020_1181_MOESM1_ESM.docx]

*Table S1 – Spatial correlation with respect to (w.r.t.) template*

| ***#IC*** | ***RSNs*** | ***Max correlation***  ***w.r.t. template*** |
| --- | --- | --- |
| *IC1* | *rDAS* | *0.2543* |
| *IC6* | *rDAS* | *0.2094* |
| *IC9* | *lDAS* | *0.2032* |
| *IC2* | *SMN* | *0.2277* |
| *IC3* | *dDMN* | *0.2810* |
| *IC11* | *vDMN* | *0.2046* |
| *IC14* | *vDMN* | *0.3669* |
| *IC16* | *aDMN* | *0.4381* |
| *IC5* | *AN* | *0.2719* |
| *IC12* | *LN* | *0.1605* |
| *IC13* | *DAN* | *0.2181* |
| *IC15* | *mPV* | *0.2505* |
| *IC19* | *SN* | *0.2000* |
